# Motion capture device reveals a quick learning curve in vascular anastomosis training

**DOI:** 10.1007/s00595-023-02726-5

**Published:** 2023-07-19

**Authors:** Shota Tsuyuki, Kazuhiro Miyahara, Katsuyuki Hoshina, Takuya Kawahara, Masamitsu Suhara, Yasuaki Mochizuki, Ryosuke Taniguchi, Toshio Takayama

**Affiliations:** 1 Department of Vascular Surgery, Graduate School of Medicine, The University of Tokyo, 7-3-1, Hongo, Bunkyo-Ku, Tokyo, 113-8655 Japan; 2 Clinical Research Promotion Center, The University of Tokyo Hospital, Tokyo, Japan

**Keywords:** Suturing, Vascular anastomosis, Off-the-job training system, Education, Leap Motion Controller

## Abstract

**Purpose:**

Surgical procedures are often evaluated subjectively, and an objective evaluation has been considered difficult to make and rarely reported, especially in open surgery, where the range of motion is wide. This study evaluated the effectiveness of surgical suturing training as an educational tool using the Leap Motion Controller (LMC), which can capture hand movements and reproduce them as data comprising parametric elements.

**Methods:**

We developed an off-the-job training system (Off-JT) in our department, mainly using prosthetic grafts and various anastomotic methodologies with graded difficulty levels. We recruited 50 medical students (novice group) and 6 vascular surgeons (expert group) for the study. We evaluated four parameters for intraoperative skills: suturing time, slope of the roll, smoothness, and rate of excess motion.

**Results:**

All 4 parameters distinguished the skill of the novice group at 1 and 10 h off-JT. After 10 h of off-JT, all 4 parameters of the novices were comparable to those of the expert group.

**Conclusion:**

Our education system using the LMC is relatively inexpensive and easy to set up, with a free application for analyses, serving as an effective and ubiquitous educational tool for young surgeons.

**Supplementary Information:**

The online version contains supplementary material available at 10.1007/s00595-023-02726-5.

## Background

The educational system in place to teach technical skills to inexperienced surgeons is mainly based on subjective evaluations by senior surgeons, with recommendations including the Operative Performance Rating System (OPRS) [1–3]. Until now, few widely accepted evaluation methods other than the length of procedure time have been reported [4]. Hand movement is one of the most important factors for surgeons and is an assessment item used in most OPRSs. If hand movements can be objectively analyzed, young surgeons could imitate the movements involved in skilled techniques and receive feedback on their own procedures.

**Fig. 1:**
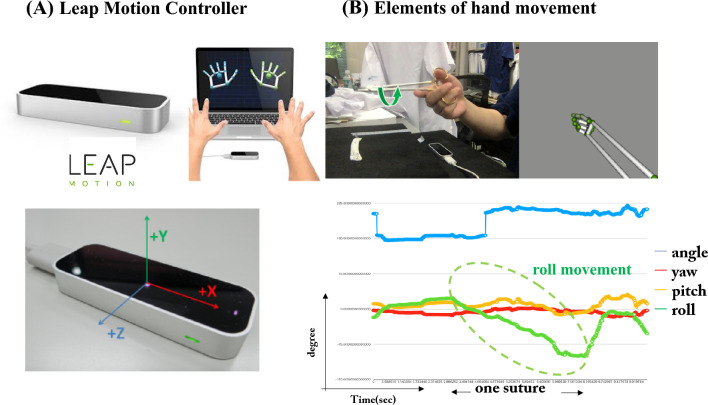
**A** The Leap Motion Device is a palm-sized device (3 × 8 × 1 cm) that is equipped with an infrared camera that captures the hands and fingers illuminated by the light-emitting diode. The device analyzes the position of the hands and fingers in three-dimensional space. **B** Hand movements were analyzed using four elements: roll, pitch, yaw, and wrist angle

The Leap Motion Controller^™^ (LMC) (Ultraleap, Inc., Mountain View, CA, USA) is a portable, low-cost motion capture device designed to track joint positions, including the wrist and fingers, with an accuracy of 1/100 mm three-dimensionally [5, 6] (Fig. 1A). We have been using this device as an educational tool for suturing skills and have previously reported that the “roll” movement effectively reflects the described skills [7]. In addition, we have integrated an off-the-job training (Off-JT) system (dry laboratory) in our department for medical students and young surgeons [4].

This study compared the scores of medical students after 1 and 10 h of Off-JT and those of vascular surgeons based on data derived from the LMC.

## Methods

### Setting of suture training

Since the Japanese Board of Cardiovascular Surgery mandated 30 h of Off-JT for new cardiovascular specialist applicants for board certification in 2017, we have devised various training methods for suturing prosthetic grafts [4, 8, and 9]. The novice operators initially start training by learning how to handle surgical devices (such as needle holders, forceps, and scissors) and suturing large (14–20 mm diameter) prosthetic grafts on a table. Trainees are subsequently made to suture smaller (1–8 mm diameter) grafts. During training, they learn various kinds of suturing techniques, including end-to-end, end-to-side, and parachute anastomotic methods. Finally, they are allowed to anastomose one graft to another fixed at the bottom of a 20-cm-deep plant pot [4]. Throughout the training, the applicants are tutored and supervised by experienced vascular surgeons of our department.

### Elements of hand movements

We previously described that hand movement is composed of four elements: roll (motion along the longitudinal axis), pitch (motion in the vertical plane), yaw (motion in the horizontal plane), and wrist angle (angle between the wrist and forearm) (Fig. 1B). In the present study, the captured data of the hand movements were converted into a graph of time and angle of the elements via the LMC. Among the four elements, only “roll” showed a significant difference between the novice and expert groups [7] (Table 1); we therefore chose it to evaluate training proficiency.

**Table 1:** Comparison of the “roll” parameters for the training time of the novices and the specialists’ parameters

	Novice after 1 h training (*n* = 50)	Novice after 10 h training (*n* = 6)	Specialist (*n* = 6)	*p* value	
				1 h vs. 10 h training	10 h vs. Specialist
(1) Suturing time	10.44 ± 0.70	5.31 ± 0.58	4.43 ± 0.65	0.007	0.337
(2) Slope of roll	16.09 ± 1.46	27.48 ± 2.70	30.91 ± 3.76	0.009	0.476
(3) Smoothness	0.63 ± 0.04	0.93 ± 0.03	0.96 ± 0.02	0.010	0.412
(4) Extra-movement rate	31.62 ± 3.11	4.00 ± 3.35	2.40 ± 1.96	0.019	0.689

*T* test, (± SE)

### Setting of motion capture with LMC

**Fig. 2:**
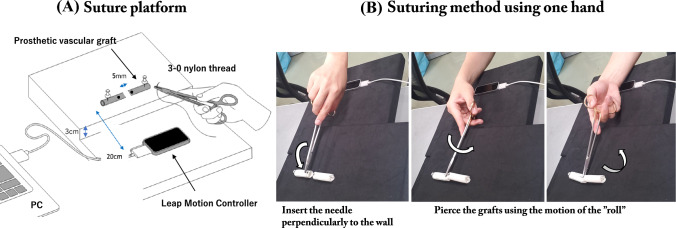
**A** Setting of the Leap Motion Controller, laptop, and suturing of the prosthetic graft. **B** Suturing method. The needle is perpendicularly inserted in the graft wall and pierced smoothly using roll movement

We placed two prosthetic grafts fixed on a board in an end-to-end fashion with a 5-mm distance between them. A dot was marked on each end of the graft as a guide for needle entry. The board was placed at a level 3 cm higher than the LMC, and the grafts were set 20 cm away from the LMC (Fig. 2A). The participants pierced the curved needle from the dot of the right graft to that of the left graft (Fig. 2B). Suturing was performed with a single hand only. Since only the outer end of the graft was pinned to the cork board, the graft could easily move when the motion was crude and unsmooth, especially when the needle point was not perpendicular to the graft. Without the support of the other hand, accurate and smooth movement with appropriate hand rotation is needed. The procedure time and motion captured using the LMC were analyzed (Video 1).

### Parameters used to evaluate roll movement

**Fig. 3:**
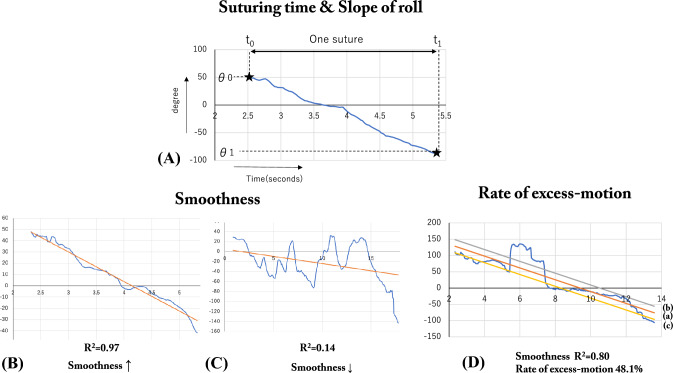
Figure of all four parameters. **A** Changes in the degrees of roll in one suture. **B** shows a better smoothness score compared to (**C**). **D** shows a good smoothness score; however, the graph showed “excess motion,” indicating a lack of proficiency

To evaluate the proficiency of the suturing hand motion, we determined new parameters in addition to procedural (suturing) time. We considered one suturing motion as the time interval from the maximum value of the “roll” (*t* = *t*_0_) to that of the minimum value (*t* = *t*_1_). Subsequently, suturing time (1) was calculated as *t*_1_ – *t*_0_. Next, the difference in the degree (*θ*) between two piercing points (*t*_0_ and *t*_1_) was calculated as |*θ*_1_ – *θ*_0_|, and the slope of the roll (2) was calculated as |*θ*_1_ − *θ*_0_|/|*t*_1_ − *t*_0_|, defined as the angular velocity of wrist rotation (Fig. 3A). Smoothness (3) was set as an indicator that the sequence of movements in the procedure was “stable” and defined using the coefficient of determination in statistics.

$$ R^{2}  = 1 - \frac{{\sum\limits_{{i = 1}}^{n} {\left( {y_{i}  - \hat{y}_{i} } \right)^{2} } }}{{\sum\limits_{{i = 1}}^{n} {\left( {y_{i}  - \overline{y} } \right)^{2} } }} $$ (Fig. 3B, C)

The coefficient of determination is a value ranging from 0 to 1. The higher the value, the smaller the variation of the graph, indicating a greater procedure stability.

Even among participants with a high smoothness score, novices occasionally made sudden and erratic movements. To assess such irregular movements, we determined the rate of excess motion (Fig. 3D). In detail, we drew a simple regression line (*Y* = *aX* + *b*) to illustrate the change in roll in one suture (red line (a) in Fig. 3D) and subsequently used “confidence intervals” to estimate the expected range of changes in roll. Confidence intervals were expressed as ± 3 × standard error, which was calculated as the mean standard error of the six experts (*σ*) from the regression line. The formula for the upper and lower limits of the confidence intervals was expressed as *Y* = *aX* + *b* ± 3*σ* (gray [b] and yellow [c] lines in Fig. 3D). Finally, the sum of the time outside the confidence interval per suture time was obtained and defined as the “rate of excess motion.”

### Participants

We recruited 50 medical students from the University of Tokyo (novice group) and 6 vascular surgeons with more than 15 years of clinical experience (expert group). Initially, these 50 participants in the novice group underwent suturing training, including guidance on device handling and a technical introduction for 1 h. Thereafter, the suturing motions of both groups were captured using the LMC, and the data were analyzed and compared between the groups. Six novices underwent an additional 10 h of surgical suture training. Concomitantly, six experts were briefed on the suturing method without any off-JT. After confirming the dispersion of the parameters, we compared and analyzed the data of the six novices and six experts.

The study protocol was approved by the Institutional Research Ethics Committee of The University of Tokyo Hospital (approval number: 11567).

### Statistical analyses

The data were analyzed using the Microsoft^®^ Excel^®^ software program, version 16.59 ([Microsoft Corporation One Microsoft Way Redmond, WA 98052-7329 USA]). Group differences were evaluated using the paired and unpaired *t* test for continuous variables, and the correlation among items was determined using a simple linear regression analysis. A *p* value < 0.05 was considered statistically significant. Continuous values are expressed as the mean ± standard error.

## Results

### Comparing the parameters after 10 h of off-JT

**Fig. 4:**
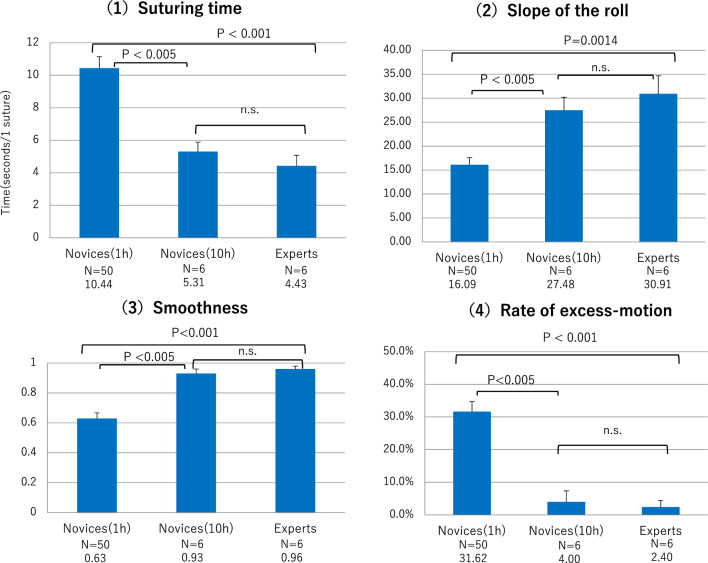
The comparison of (1) suturing time, (2) slope of the roll, (3) smoothness, and (4) rate of excess motion between the novice group at 1 and 10 h off-JT and those of the expert group

Six of the 50 novices underwent additional practice and received a total of 10 h of Off-JT. Their data were subsequently captured and analyzed again. Compared with all novices, the 6 novices who underwent 10 h of training showed significantly improved parameters, including a significantly shorter suture time (5.31 ± 0.58 vs. 10.44 ± 0.70 s; *p* < 0.005), larger degree of the slope (27.48° ± 2.70°/s vs. 16.09° ± 1.46°/s; *p* < 0.005), smoother motion (closer to a value of 1; 0.93 ± 0.03 vs. 0.63 ± 0.04; *p* < 0.005), and smaller rate of excess motion (4.00% ± 3.35% vs. 31.62% ± 3.11%; *p* < 0.005) (Fig. 4).

### Differences between the novice and expert groups

After 1 h of off-JT, the expert group demonstrated a significantly shorter suture time than the novice group (4.43 ± 0.65 vs. 10.44 ± 0.70 s; *p* < 0.001), a larger degree of slope (30.91° ± 3.76°/s vs. 16.09° ± 1.46°/s; *p* = 0.0014), better smoothness, with a value close to 1 (0.96 ± 0.02 vs. 0.63 ± 0.04; *p* < 0.001), and smaller rate of excess motion (2.40% ± 1.96% vs. 31.62% ± 3.11%; *p* < 0.001).

Furthermore, we compared the scores of the 6 novices after 10 h of training with the experts’ scores. Surprisingly, all four parameters of the novices statistically reached a level comparable to that of the expert group (Fig. 4).

## Discussion

The values of all four parameters after one hour of training were significantly different between the novice and expert groups. Furthermore, after 10 h of training, the values of all 4 parameters for the 6 novices had dramatically improved and were comparable to those of the expert group.

In our previous study, we demonstrated the usefulness of the LMC as an educational tool for anastomosis training for surgeons [7]. There are various tools and systems to track a surgeon’s procedural skills and provide educational feedback [10–13]; however, a majority of them are too heavily equipped and may be too expensive to be implemented easily in all facilities. We focused on inexpensiveness (cost: approximately $100 USD) and simplicity and conducted a study on anastomotic evaluations using an LMC. We introduced the novel setting of graft anastomosis, which can easily reveal differences in surgical skills and new parameters, thus being able to represent the operator’s skills. Furthermore, we evaluated the novice group after 10 h of Off-JT and found that 10 h of training brought the Off-JT performance of the novice group remarkably close to that of the expert group.

The motion tracking of the surgical procedure is likely to be used in laparoscopic or robotic surgery because the hand motion, which is linked to the device movement and its area, was limited, making it easier to analyze the motion pattern [10, 11]. The Advanced Dundee Endoscopic Psychomotor Tester is a system that incorporates a dual-gimbal mechanism, which accepts two endoscopic instruments in a surgical procedure. The system revealed that three parameters—instrument error contact time, execution time, and task completion score—were better in master surgeons than in trainees [10]. However, this system can only evaluate movement and cannot provide feedback to trainees. iSurgeon, the sensor- and expert model-based training system, can provide trainees with real-time feedback based on expert motion models. Thus, this system may be the ideal motion caption-based feedback system [11].

In robotic surgery, it is easier to capture motion three-dimensionally than various other surgical modalities [12]. However, the area of hand motion during open surgery should be wide. The Imperial College Surgical Assessment Device (ICSAD) is a combination of a commercially available electromagnetic tracking system and associated software [13]. In the study of Datta et al., the operator attached a single 10-mm electromagnetic tracker to the dorsum of the hand, allowing the system to collect the three-dimensional paths of hand movement. Interestingly, a previous study evaluating suture movement using ICSAD demonstrated that with increased proficiency, procedural time and number of movements decreased, but the path length did not change. In comparison, our study set of four parameters represented the operators’ proficiency more accurately since all parameters showed improvement with additional training.

These four factors might not be completely independent of each other. Although the suturing time and slope of the roll were inversely related, time is the most universally used and easily understood objective factor; we therefore set it as an evaluating factor. Smoothness refers to a rhythmic pattern of acceleration and deceleration of movement, and this factor should be evaluated to represent jerk minimization [12]. In our study, we defined it according to the degree of motion deviation during one suture. However, we found that some novice operators made sudden and large jerks that could not be represented with smoothness. The causes of excess motion possibly include hesitant movements that prevented the needle from being inserted perpendicularly into the graft or excessive force in the device grasp due to an inability to relax. These two factors are also not statistically independent; however, we have not yet established a method to evaluate these movements by a single factor.

Suturing with a single hand is a unique training trial. We found that if the needle is stitched perpendicularly, the graft can be penetrated smoothly without the support of the other hand. Unlike the gastrointestinal tract, anastomosis of blood vessels requires the needle to be inserted perpendicular to the vessel wall to evert them. In actual open surgery of the abdominal aorta, we sometimes need to use one hand to achieve an adequate surgical field and have to grasp and insert the needle using only the other free hand. Furthermore, the difference in technique between a skilled and novice surgeon becomes apparent when this suturing method is used. As the video shows, the needlepoints handled by the novice operators tended to slip inside the graft or lift the graft roughly, even if they were caught (Video 1).

We have created a free web application for the practical use of the LMC for Off-JT (https://katagraphy.web.app/). By reading the movement captured by the LMC with this application, three factors—suturing time, slope of the role, and smoothness—are calculated simultaneously. We are currently improving the application to automatically calculate the rate of excess motion, as it was difficult to do so.

We should note one limitation of this study: the selection bias of the 6 novices who underwent 10 h of training. Although no marked variability was observed in any of the four parameters of the 6 novices compared with other novices, their motivation may be considered higher than that of others, which could be a limitation of this study.

## Conclusion

In this study, we described the Off-JT system in our department and the methodology for evaluating the suturing technique using a motion capture device (LMC). We set four parameters—suturing time, slope of the roll, smoothness, and rate of excess motion—to evaluate suturing skill objectively. After 10 h of Off-JT, the skill of the novice operators improved dramatically, and all parameters were comparable to those of experts. Our LMC system, which is inexpensive and easy to set up and has a free application for analyses and evidence, can be used as an education tool for young surgeons at any institution.

### Supplementary Information

Below is the link to the electronic supplementary material.

Video 1. Movie demonstrating the analytic system using the LMC, suturing method, and examples of the procedure by a novice operator and an expert. Supplementary file1 (MP4 18611 KB)

## Abbreviations

OPRS: Operative Performance Rating System
LMC: Leap Motion Controller
Off-JT: Off-the-job training
ICSAD: Imperial College Surgical Assessment Device
